# Platelet-rich plasma administration to the lower anterior vaginal wall to improve female sexuality satisfaction

**DOI:** 10.4274/tjod.galenos.2019.23356

**Published:** 2020-02-28

**Authors:** Gökmen Sukgen, Aşkı Ellibeş Kaya, Ebru Karagün, Eray Çalışkan

**Affiliations:** 1 Private Practice Clinic, Clinic of Obstetrics and Gynecology, Adana, Turkey; 2 Düzce University Faculty of Medicine, Department of Obstetrics and Gynecology, Düzce, Turkey; 3 Düzce University Faculty of Medicine, Department of Dermatology, Düzce, Turkey; 4 Okan University Faculty of Medicine, Department of Obstetrics and Gynecology, İstanbul, Turkey

**Keywords:** Platelet-rich plasma, female sexual dysfunction, female orgasmic disorder

## Abstract

**Objective:**

To investigate the effect of platelet-rich plasma (PRP) injection to the lower one-third of the anterior vaginal wall on sexual function, orgasm, and genital perception in women with sexual dysfunction.

**Materials and Methods:**

Four sessions of PRP were administered to the anterior vaginal wall of 52 female patients with sexual dysfunction and orgasmic disorder [Female Sexual Function Index (FSFI) total score ≤26 orgasmic subdomain score ≤3.75]. Prior to the PRP administrations in each session, the FSFI validated in Turkish, the Female Genital Self-Image Scale (FGSIS), the Female Sexual Distress Scale-Revised (FSDS-R), and Rosenberg’s Self-Esteem Scale were used and in the final follow-up, and the Patient Global Impression of Improvement (PGI-I) was performed and the results were analyzed.

**Results:**

Following the application of the PRP, the total FSFI score was observed as 27.88±4.80 and the total score was 26 and above in 50% of the patients (p<0.001). Orgasm subdomain scores were found as 2.11±1.20 before the PRP treatment and 4.48±1.14 afterwards (p<0.001). A significant change was observed in all sub-domains after PRP and it was observed that this change started after the first administration (p<0.001). A statistically significant increase was determined in FGSIS genital perception scores, which was significant between the 1^st^ and 2^nd^ months (p<0.001). The FSDS-R scores showed a minimal increase in stress scores as the application number increased, but a statistically significant decrease was observed in the 4th administration (p<0.001). No statistically significant difference was found in Rosenberg Scale scores before and after treatment (p=0.389). High satisfaction was found in PGI-I scores.

**Conclusion:**

As a minimally invasive method, PRP administration to the distal anterior vaginal wall may improve female sexuality with high satisfaction.

**PRECIS:** PRP administration to the lower anterior vaginal wall to improve female sexuality.

## Introduction

Platelet-rich plasma (PRP) treatment aims to increase the self-healing ability of the human body by increasing neovascularization and collagen formation through the effect of high concentration autologous growth factors administered to the tissue^(1)^. The most important advantages are it being autologous and reliable^(2,3)^. Case series, pilot studies, and case reports related to gynecologic use of PRP are observed in the literature^(2,3)^. PRP has been used in atrophic diseases such as lichen sclerosis in the vagina, stress urinary incontinence, episiotomy scars, and lubrication disorders in the vagina^(2)^. The first use for sexual dysfunction in women was performed by Charles Runels under the name of O-Shot^(3)^. Improvement of sexual functions was reported with PRP administration to the G-spot. Sexual dysfunction classification systems are structured on the coordination of the phases including the desire, arousal and orgasm of the sexual response cycle, which is defined by Master and Johnson and developed by Kaplan^(4)^. According to the criteria of the Diagnostic and Statistical Manual of Mental Disorders-Fifth Edition (DSM-5), desire/arousal and orgasmic disorders are the most common causes of female sexual dysfunctions^(5)^. Sexual stimulation of women is known to be provided mostly by touching^(6)^. The most sensitive point to stimulus in the female body is the genital area^(6)^. In the vagina, the lower one-third of the anterior region has been proved to have more nerves immunohistochemically^(7,8,9)^ and it is known that the response of the distal anterior vaginal wall to contact and to pressure is higher than the other part of the vagina, in penis-vagina penetration^(6,10)^. PRP treatment has the potential to be part of a surgical and non-hormonal approach in patients with sexual dysfunction with regenerative changes by increasing collagen formation and neovascularization in the anterior vaginal wall. The aim of this study was to investigate the effect of PRP injections to the lower anterior vaginal wall on sexual function, orgasm, and genital perception of patients with sexual dysfunction.

## Materials and Methods

Our retrospective cross-sectional study was performed on 52 patients who fulfilled the criteria for inclusion and exclusion and who were admitted to a private clinic due because of sexual dysfunction between December 2017 and April 2019 and administered vaginal PRP.

### The inclusion criteria of our study included

Patients aged over 18 years of age, who had been sexually active for the last 12 months and had had sexual intercourse at least every 15 days, dyspareunia, who could not achieve orgasm, had difficulty in orgasm, had dryness and pain in intercourse in the last six months, absence of sexual appetite, those who met the diagnostic criteria of female sexual interest/arousal disorder or orgasmic disorder according to the DSM-5, and patients with a total of 26.5 and six points and whose orgasm subdomain is below 3.75 in the Female Sexual Function Index (FSFI) questionnaire completed in the first admission, were included in the study. Patients who were sexually inactive, who had organic pathology in vaginal examination, who were pregnant, aged under 18 or over 55 years, postmenopausal, using antidepressant-psychotropic drugs, using sexual strengthening medication such as sildenafil, using topical estrogen in the past year, using the contraceptive pill, alcohol and drug addicts, those who had undergone vaginal aesthetic and genital oncological surgery, receiving chemotherapy or radiotherapy, those whose FSFI questionnaire scores were above 26.5, and those with orgasm subdomains above 3.75 were excluded from the study. Primary inorgasmic patients were excluded from the study. The patients were asked whether their spouses had erectile dysfunction and premature ejaculation; respondents who said “yes” were excluded from the study. The Beck Depression Inventory was applied to the patients who met the inclusion criteriai and patients with a score of 17 and above were excluded from the study.

### Scales

The following questionnaires were administered before the PRP administration in the 1^st^, 2^nd^, 3^rd^, and 4^th^ month and at the 6^th^ month follow-up. The validated Turkish version of the FSFI, Female Genital Self-Image Scale (FGSIS), Female Sexual Distress Scale-Revised (FSDS-R), and the Rosenberg Self-Esteem Scale (RSES) were applied to all participants^(11,12,13,14)^. The Patient Global Impression of Improvement (PGI-I) was completed at the 6^th^ month for the evaluation of the patient’s satisfaction. The FSFI is a brief instrument for the assessment of sexual function that consists of 19 questions and has been validated based on DSM-4 diagnoses of desire disorder, arousal disorder, and orgasmic dysfunction. Questions are scored for domains of libido, arousal, lubrication, orgasm, satisfaction, and pain^(15)^. The cut-off scores for the FSFI scales were created by using the scale-specific means for women without sexual dysfunctions minus one standard deviation (SD) as reported by Wiegel et al^(16)^. The cut-off scores were as follows: 3.16 for desire, 3.97 for arousal, 4.31 for lubrication, 3.75 for orgasm, 3.85 for sexual satisfaction, and 4.22 for pain. An FSFI total score of 26.55, out of a maximum possible score of 36, is generally considered to be the optimal cut-off score to differentiate women with and without sexual dysfunction^(16)^. The seven-item FGSIS assesses women’s feelings and beliefs about their own genitals and has established reliability and validity in a convenience sample^(17)^. Respondents’ scores on each item were summed for a total sum score ranging from 7 to 28, with higher scores indicating a more positive genital self-image. The FSDS-R assesses different aspects of sexual-activity-related distress in women. The total score, ranging from 0 to 52, can be computed by adding all 13 item scores. Higher scores indicate higher levels of sexual distress^(13)^. The RSES, developed by Rosenberg, is one of the most widely used tools to assess global self-esteem by measuring both positive and negative individual feelings^(18)^. The RSES is a 10-item, 4-point Likert-type Scale, ranging between 0 and 30. Scores between 15 and 25 are within the normal range, and scores above 25 are indicative of high self-esteem. PGI-I is a global index that may be used to rate the response of a condition to therapy. It is a simple, direct and easy-to-use scale that is intuitively understandable to both physicians and patients, and has a single question for comparing now and before beginning the application on a scale from 1: very much better, to 7: very much worse.

### Application

The patients were informed on how the PRP application would be performed, together with the possible risks and complications. The procedure was performed after written informed consent was obtained.

The patients were placed on the operating table in the dorsal lithotomy position when the bladder was empty, half an hour before the procedure for applying a local anesthesia cream which contained lidocaine 2.5% and prilocaine 2.5%. The local anesthetic was administered around the clitoris and the vaginal lower one-third of the region.

A PRP kit was used for the PRP administration. The PRP kit consists of 2 PRP tubes, re-suspension tube, 2 injectors, and 2 needles in a single sterile mold. Each PRP tube has a volume of 10 mL and contains 1 mL of citrate.

The PRP+public-private partnership (PPP) preparation steps in our study were as follows:

i.  Approximately 18 mL of venous blood from the cubital region of the upper extremity of the patients were taken to two special vacuumed CE-certified PRP tubes;

ii.  The collected blood was centrifuged at 3200 rpm for eight minutes;

iii.  After centrifugation, it was found that the plasma (upper layer), platelets and leukocytes (middle layer called “Buffy coat”) and erythrocytes (lowest layer) were divided into three layers;

iv.  Approximately 2 mL of PRP and 2-3 mL PPP from each tube was transferred to the re-suspension tube;

v.  For a more homogeneous spread of platelets in the re-suspension tube, the tube was shaken gently by hand for 30 sec;

vi.  After isolation of the 4-5 mL of PRP+PPP from the re-suspension tube, calcium chloride (0.5 mL) was added, thereby activating the thrombin cascade, causing degranulation of platelets, releasing growth factors and cytokines, and starting the transformation to the platelet-rich fibrin matrix;

vii.  A total of 9 mL PRP+PPP mixture was obtained from both tubes;

vii.  After the CaCl was added, the treatment was performed within 5 min;

ix.  The PRP+PPP was now ready for injection;

x.  PRP+PPP injected using a 21 gauge (G) needle.

**Figure 1:**
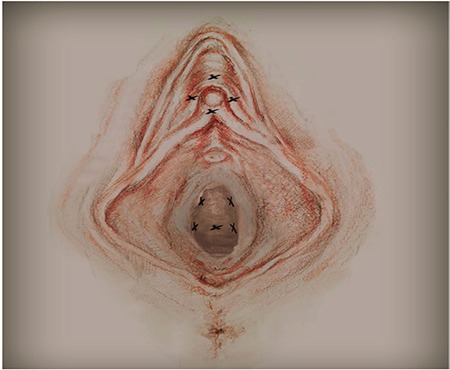
The application areas to the lower anterior vaginal wall and around the clitoris.

Patients were placed the lithotomy position again and PRP was administered to form pili of 4 cc around the clitoris in the direction of clock positions of 12, 3, 6, and 9, each with 1 cc, 2 cc subcutaneously; right/left of paraurethral vaginal wall each with 1 cc, 3 cc; and mid-urethral midline/right/left 1 cc. The PRP applications were administered using 31-G needles (Figure 1).

This administration was continued once every four weeks, for four months. The patients were evaluated by repeating the questionnaires in each administration and at the 6^th^ month.

## Results

**Figure 2:**
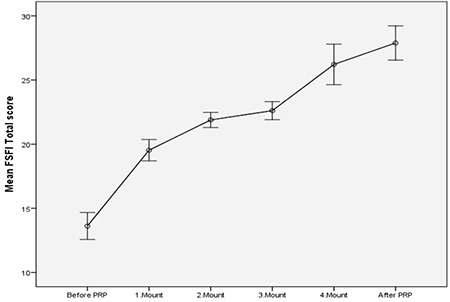
Graphs of Mean Female Sexual Function Index Total score at application times. Bars are standard deviation. After PRP; 6^th^ month checks are shown. FSFI: Female Sexual Function index, PRP: Platelet-rich plasma

**Figure 3:**
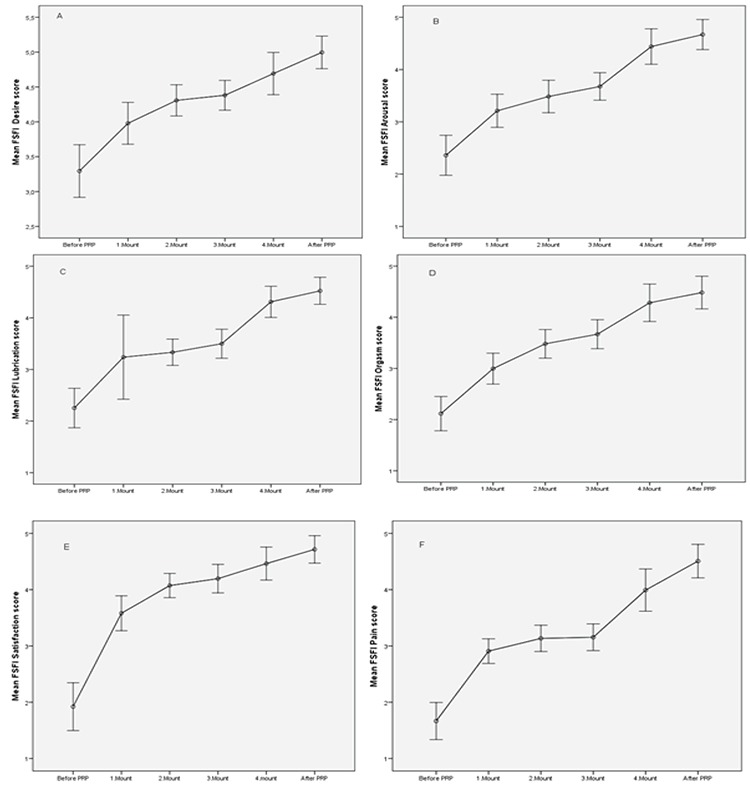
Graphs of mean Female Sexual Function Index (FSFI) subdomain scores at application times Mean FSFI desire score, B. Mean FSFI arousal score, C. Mean FSFI lubrication score, D. Mean FSFI orgasm score, E. Mean FSFI satisfaction score, F. Mean FSFI pain score. Bars are standard deviation. After PRP; 6^th^ month checks are shown.

**Figure 4:**
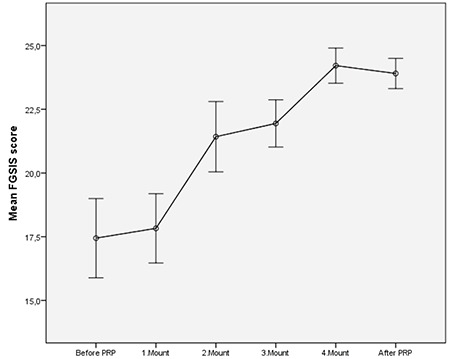
Graphs of mean the Female Genital Self-Image Scale Total score at application times. Bars are standard deviation. After platelet-rich plasma; 6^th^ month checks are shown.

**Figure 5:**
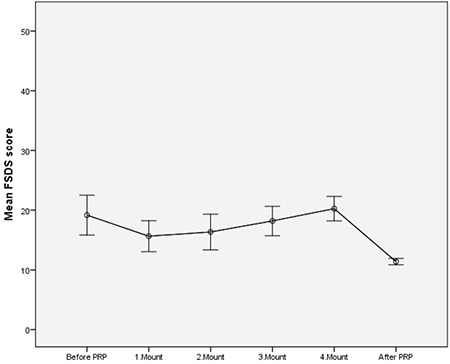
Graphs of Mean the Female Sexual Distress scale-revised Total score at application times. Bars are standard deviation. After PRP; 6^th^ month checks are shown. PRP: Platelet-rich plasma

The mean age of the patients was 37.5±9.8 (mean ± SD; 22-55 minimum-maximum) years. The mean body mass index was 26.54±5.10 (mean ± SD; 18.29-40.00 minimum-maximum) kg/m^2^, 100% wer sexually active, 21% were single, and 79% were married. The results of the PRP before, during, and after the administration for 4 months are seen in Table 1 and Figures 2, 3, 4, 5. The pre-treatment FSFI total score was 13.61±3.78 (mean ± SD), and the total score of all patients was below 26. The subdomains of FSFI of desire, arousal, lubrication, orgasm, satisfaction and pain mean scores (mean ± SD) were found as 3.29±1.35, 2.35±1.37, 2.25±1.37, 2.11±1.20, 1.92±1.52, and 1.66±1.18, respectively. The pre-treatment FGSIS scores were 17.44±5.58 (mean ± SD), FSDS-R scores were 19.17±12.00 (mean ± SD), and RSES scores were found as 20.96±6.08 (mean ± SD) (Table 1).

After application of the PRP, the total score of FSFI was 27.88±4.80 (mean ± SD) and the total score was 26 and over in 50% of cases. The increase in the total score was found statistically significant after the 1^st^ month (p<0.001, Figure 2). After PRP, the subdomains of FSFI of desire, arousal, lubrication, orgasm, satisfaction, and pain mean scores were found as (mean ± SD); 4.99±0.83, 4.66±1.03, 4.52±0.94, 4.48±1.14, 4.71±0.87, and 4.50±1.06, respectively. A significant initial change in all sub-domains was observed after the first administration (p<0.001, Figure 3). The FGSIS scores after treatment were 23.90±2.12 (mean ± SD), FSDS-R scores were 11.38±1.91 (mean ± SD), and RSES scores were 22.05±5.70 (mean ± SD) (Table 1, Figures 2, 3, 4, 5). No statistically significant difference was observed in the Rosenberg scale before and after treatment (p=0.389).

A statistically significant increase was determined in FGSIS genital perception scores, which was prominent between the 1^st^ and 2^nd^ months (p<0.001, Figure 4). FSDS-R scores showed a minimal increase in the stress score as the application number increased, but a statistically significant decrease was observed between the 1^st^ and 4^th^ months (p<0.001, Figure 5). As a result, it was seen that there was a significant improvement in scale scores after four PRP administrations, except on the RSES.

## Discussion

In this study, we aimed to investigate the effect of PRP injection to the lower anterior vaginal wall on sexual function, orgasm, and genital perception of patients with sexual dysfunction. With significant changes in the results of the survey, we found that a positive effect started in the first session of PRP. We found an increase in the satisfaction of the patients after the procedure. PRP is based on the separation of a small amount of blood taken from the patient into a special tube, after which centrifugation is performed and the obtained “PRP” is returned to the same patient by injection. PRP has been used in many areas such as cosmetic use, wound healing, and urologic and orthopedic applications^(1,2,3)^. In many medical diseases, it is becoming increasingly popular among minimally invasive methods with a wide area of use. It is known that PRP increases collagen formation and neovascularization with growth factors (platelet-derived growth factor, transforming growth factor-b, epidermal growth factor) by 5-10 times more than normal blood. PRP therapies aim to increase the self-healing ability of the human body due to the effects of high-concentration autologous growth factors applied to the tissue^(1)^. In the literature, it is observed that PRP use in gynecology is less than in other disciplines, and is limited to case series, pilot studies, and case reports^(2,3)^. It has been used in atrophic diseases such as vaginal lichen sclerosis, stress urinary incontinence, episiotomy scars, and lubrication disorders^(2,19)^. PRP has been found to be effective in mesh erosion; PRP-coated mesh treatment has been revealed by *in vitro* and animal studies to result in better wound healing, increased synthesis of collagen 3, and neovascularization^(20,21)^.

In studies investigating major motivators for genital aesthetic operations, notably labioplasty, it is seen that the expectation of an “increase in sexual function” is up to 50%^(22,23)^. In other words, to increase sexual functions, half of these patients have operations in which they may experience complications. In addition, the treatment of female sexual dysfunction is limited to psychotherapy and hormonal support in A-level^(5)^. For this reason, less invasive and low adverse-risk interventions may also be planned through good identification of the major motivators for patients whose only expectations are to increase sexual functions. From this point of view, laser, filler injections, and PRP are gaining popularity nowadays^(3,24,25,26)^. It is also possible that in such minimally invasive methods, a positive effect is enhanced by providing the woman’s sensitive focus to the vagina during sexual intercourse. The first use for sexual dysfunction in women was performed by Charles Runels under the name of O-Shot™^(3)^. Improvement of sexual functions was reported with PRP administration to the G-spot and glans clitoris. In this pilot study, PRP was administered to two regions, the clitoris and the anterior vaginal wall, into a space between the vagina and urethra most distal from bladder. However, the lack of a precise standardization of the location of the G-spot suggests that the administration area would result in a low response to a single injection.

In a human study, significantly increased density of nerves and microvessels in the distal one-third of the anterior vaginal wall were seen^(9)^. The anterior wall, compared with the posterior wall and the distal part of the anterior, has been shown to have more nerves immunohistochemically^(7)^. In a cadaver study, it was found that the second one-fifth partition of the distal anterior wall had significantly richer innervation than the surrounding areas^(8)^.

The name G-spot, G-spot neurovascular complex, anterior wall erogenous complex, clitorovaginal-urethrovaginal complex, or whichever term it is called, is the female distal anterior vaginal wall, which is known to be more sensitive. The response of the distal anterior vagina, which is more sensitive, to penis-vagina penetration is higher^(6,10,27)^. Based on this, we planned to adminster our injections more commonly in the distal anterior one-third vagina region. We administered five injections in the vaginal wall. In previous studies, PPP has been shown to be as effective as PRP and to trigger angiogenesis and wound healing^(28,29)^. After centrifugation, we used the plasma part of the poor platelet in order to be able to benefit from the growth factors within it and due to our wide range of applications. It is known that increased blood flow through the clitoris is correlated with improved sexual function in women^(30)^. Accordingly, we administered four injections to the clitoris and its surroundings.

There is no consensus in the literature about how many times and how often PRP administrations should be performed. Runels et al. made one single application for sexual dysfunction and they expressed positive results. In our study, we performed four administrations, but we found a significant change in total and all subdomains of FSFI after the first application. As the repetitions of the application increased, the acceleration of the positive effect decreased, but the increase continued. Therefore, the frequency of PRP and the number of repetitions can be planned according to the patient’s condition.

In our study, FGSIS scores were found significantly lower than the mean values in the original study. This may be due to the fact that the selected population were patients with sexual dysfunction because the positive correlation between FSFI and FGSIS is known^(12,17)^.

In our study, a significant decrease was observed in FSDS-R scores with the fourth administration. This shows that the administration results in a decrease in sexual distress. However, a negligible increase was observed in sexual distress after the first application until the third application. This may be the result of anxiety caused by expectation.

PGI-I scores, which we applied in the 6^th^ month in order to evaluate the satisfaction of the patients, were found to be high, thereby supporting the positive increase in the survey scores.

To date, no adverse effect have been reported in vaginal applications in the literature. This is explained by the fact that the contents of PRP are from the patient’s own body. In our study, no adverse effects of the administration were observed in any patients.

### Study Limitations

The small number of studies in the literature on PRP and sexual dysfunction and the high number of patients in our study compared with other studies are strengths of our study. Furthermore, as far as we know, no similar study evaluating the efficacy of PRP in terms of orgasm is available in the literature. The lack of randomization and the lack of a control group to be performed with a placeboand retrospective design are the limitations of the study. Sexual dysfunction of partners in female sexual dysfunction is very important and the fact that this has not been evaluated objectively another limitation of our study.

## Conclusion

PRP is a minimally invasive method, which is easy to apply and fast, and has almost no adverse effects owing to it being autologous. Administration to the lower anterior vaginal wall may improve female sexuality with high satisfaction.

## Figures and Tables

**Table 1:**
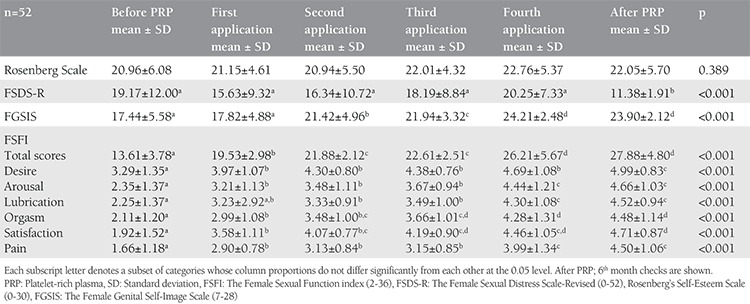
Analysis of the questionnaires, stratified by duration: before platelet-rich plasma, during and after platelet-rich plasma
